# Clinical Characteristics and Early Evolution of Neonates with Perinatal Gram-Negative Bacterial Infections

**DOI:** 10.3390/children12121692

**Published:** 2025-12-13

**Authors:** Mihaela Zaharie, Aniko Maria Manea, Marioara Boia, Oana Costescu, Daniela Cioboata, Timea Brandibur, Daniela Iacob

**Affiliations:** 1 Ph.D. School, Faculty of Medicine, “Victor Babes” University of Medicine and Pharmacy Timisoara, 2 E. Murgu Sq., 300041 Timisoara, Romania; mihaela.zaharie@umft.ro; 2 Department of Neonatology and Puericulture, “Victor Babes” University of Medicine and Pharmacy Timisoara, 300041 Timisoara, Romania; boia.marioara@umft.ro (M.B.); costescu.oana@umft.ro (O.C.); timea.brandibur@umft.ro (T.B.); iacob.daniela@umft.ro (D.I.); 3 Neonatology and Preterm Department, “Louis Ţurcanu” Children Emergency Hospital, 300011 Timisoara, Romania; cioboata.daniela@umft.ro; 4 Clinic of Neonatology, “Pius Brinzeu” County Clinical Emergency Hospital, 300723 Timișoara, Romania

**Keywords:** neonatal infections, Gram-negative bacteria, perinatal sepsis, clinical outcomes, empirical antibiotic therapy, tertiary neonatal care

## Abstract

**Background/Objectives**: Perinatal infections caused by Gram-negative bacteria pose a significant risk to neonatal health, especially in low-resource settings. These infections often lead to severe complications due to diagnostic and therapeutic challenges. This study aimed to clinical characterize, early outcomes, and risk factors associated with Gram-negative infections in neonates admitted to the tertiary neonatal intensive care unit (NICU) at “Louis Turcanu” Children’s Hospital, Timisoara. **Methods**: A retrospective, case–control study was conducted at a tertiary neonatal care unit in Timișoara, Romania, including neonates with microbiologically confirmed Gram-negative infections (*n* = 44) and a matched control group without infection (*n* = 47). Clinical, laboratory, and microbiological data were analyzed. Statistical comparisons and logistic regression analyses were used to evaluate risk factors and outcomes. **Results**: Male sex (79.5% in infected vs. 57.4% in controls; *p* = 0.0418) and vaginal delivery (43.2% vs. 17.0%; *p* = 0.00001) were significantly associated with infection. Respiratory distress (72.7%) and digestive symptoms (75.0%) were common in infected neonates. C-reactive protein and procalcitonin levels were markedly elevated in infected infants. Escherichia coli was the most common pathogen, with multidrug-resistant strains observed in bloodstream infections. Mechanical ventilation was required in 75% of infected neonates compared with 16.2% in controls (*p* < 0.0001). Mortality was higher among infected neonates (25% vs. 4.3% in the control group), although not statistically significant. **Conclusions**: Gram-negative perinatal infections are associated with considerable morbidity, particularly in male neonates and those delivered vaginally. Early identification, antimicrobial stewardship, and intensive respiratory support are essential to improving outcomes in this vulnerable population.

## 1. Introduction

Neonatal infections remain a health concern worldwide, especially in low- and middle-income countries, contributing significantly to newborn morbidity and mortality. Gram-negative bacteria such as *E. coli*, *Klebsiella*, and *Pseudomonas* are leading causes of early-onset sepsis. Prematurity and maternal infections are key risk factors, underscoring the need for early recognition and targeted interventions. The incidence of Gram-negative infection is 2.01 per 1000 live births worldwide [1].

Maternal health is crucial, as it is linked to maternal bacterial colonization and neonatal infections, particularly sepsis caused by Gram-negative organisms, underscoring the importance of maternal screening, especially in resource-limited settings [2]. Vertical transmission often occurs during delivery as bacteria ascend from the lower genital tract or cross ruptured membranes. Complications such as premature rupture of membranes (PROM) further increase the risk of infection. PROM allows bacterial entry, leading to neonatal pneumonia and sepsis, stressing the importance of vigilant prenatal care [3]. The neonatal immune system’s vulnerability, especially in preterm or low-birth-weight infants, necessitates tailored care strategies, as evolutionary adaptations in neonatal immunity are crucial for managing infections [4,5].

Gram-negative bacterial infections during the perinatal period pose a clinical challenge, particularly due to the difficulties associated with early diagnosis and the development of effective treatment strategies. Early symptoms are nonspecific (respiratory distress, fever or hypothermia, irritability, lethargy, poor feeding, and hemodynamic instability), complicating timely detection and intervention. This issue is even more pronounced in low-resource settings where diagnostic capabilities, such as blood culture sensitivity testing, are limited [6].

In treating these infections, a poor understanding of regional antimicrobial resistance trends often contributes to inadequate treatments, making effective management more challenging [7]. When empirical antibiotics must be used, delays in selecting effective regimens can worsen disease progression and outcomes [8]. In clinical care, local resistance data must guide the choice of empirical antibiotics. Resistance patterns among Gram-negative bacteria vary significantly, leading many healthcare providers to rely on broad-spectrum antibiotics without specific susceptibility information [6,7]. On the other hand, cefepime, a fourth-generation cephalosporin, has been reported to have broad efficacy against Gram-negative pathogens in neonates [9].

Antibiotic resistance among Gram-negative bacteria, particularly multidrug-resistant (MDR) strains, is a growing concern in neonatal care. MDR organisms are increasingly identified in neonatal units, areas previously considered safe for antimicrobial therapies [6,10].

Perinatal infections caused by Gram-negative bacteria pose a significant concern. While global studies have characterized various aspects of these infections, there is a notable lack of region-specific data on their clinical presentation and early outcomes, especially in tertiary care settings at the “Louis Turcanu” Children’s Hospital in Timisoara. This gap in local evidence hinders the development of targeted empirical treatment strategies and risk assessment protocols, particularly in settings where antimicrobial resistance patterns and healthcare practices may differ from global norms. Therefore, a local perspective is essential for tailoring interventions and optimizing neonatal outcomes.

The objective of this retrospective audit was to describe the loco-regional clinical presentation, maternal and perinatal context, microbiological profile, and early clinical course of neonates with culture-proven Gram-negative sepsis admitted to our tertiary NICU. A cohort of NICU-admitted neonates without infection, matched for gestational age and birth weight, was used solely as a contextual reference to quantify the magnitude of the clinical burden, not to test a causal hypothesis regarding outcomes, which are already well established in the literature. This research also seeks to enhance evidence-based practices in neonatal care by examining the local epidemiology and clinical context.

## 2. Materials and Methods

### 2.1. Study Design and Setting

This research employed a retrospective, observational, case–control design conducted within the Neonatology Department of the tertiary care teaching institution, “Lois Turcanu” Children’s Hospital, Timisoara, comparing neonates diagnosed with perinatal Gram-negative bacterial infections with those of a matched, uninfected control group.

This retrospective audit included all consecutive neonates with culture-proven Gram-negative sepsis admitted to our NICU during the study period. No a priori sample size calculation was performed as the number of eligible cases was determined exclusively by the total number of culture-confirmed infections available in the predefined timeframe.

The infected cohort included newborns with microbiologically confirmed Gram-negative sepsis diagnosed during the early neonatal period, primarily within the first 72 h of life. These cases were identified from hospital records over a predefined two-year period. They were selected based on laboratory confirmation of pathogens, including *Escherichia coli*, *Klebsiella* spp., and *Pseudomonas aeruginosa*.

Gestational age categories were defined as: term (≥37 weeks), late preterm (34–36 + 6 weeks), moderate preterm (32–33 + 6 weeks), and very preterm (<32 weeks), according to international standards. These categories were used to characterize the study population.

To enhance the robustness of the comparative analysis, a control group consisting of neonates admitted during the same timeframe who did not exhibit clinical or laboratory signs of infection was assembled. Controls were matched by gestational age and birth weight, ensuring comparability in baseline perinatal risk profiles. This matching process was critical in reducing confounding variables that could independently influence early neonatal outcomes. Mortality was recorded as a descriptive outcome only. No attempt was made to perform inferential testing or to estimate the effect size for mortality differences between infected and non-infected neonates, as there is no equipoise regarding the expected direction or magnitude of these differences. Comparative mortality data are therefore presented descriptively to illustrate the local clinical burden.

### 2.2. Data Collection and Inclusion/Exclusion Criteria

This study was based on clinical records from neonates admitted to the Neonatal Intensive Care Unit (NICU) of a tertiary care center in Timișoara, Romania, between January 2022 and December 2024. Two groups were selected: neonates with confirmed perinatal infections caused by Gram-negative bacteria (case group) and a NICU reference group, matched for gestational age (±1 week) and weight (±250 g), used to calibrate severity with prematurity or determinants without confirmed infection (control group). Data were systematically extracted from electronic health records and anonymized before analysis to ensure compliance with ethical standards and patient confidentiality. Given the retrospective design and the rarity of culture-confirmed Gram-negative infections, the study was not powered to detect predefined differences between groups. Comparative analyses were therefore exploratory and descriptive.

The dataset included various variables covering maternal, perinatal, and neonatal factors. Maternal data included risk factors such as preterm rupture of membranes (PROM), chorioamnionitis, or maternal fever. Neonatal data included gestational age, birth weight, sex, Apgar scores, mode of delivery, and need for resuscitation at birth. Clinical and paraclinical indicators were also recorded, including initial laboratory parameters (e.g., white blood cell count, C-reactive protein, and procalcitonin levels), microbiological findings, and requirements for respiratory or circulatory support. Therapeutic interventions and immediate outcomes were also noted, including the type and duration of antibiotic treatment, the need for mechanical ventilation, the duration of the NICU stay, and survival at discharge. All data were verified by the authors for consistency and accuracy.

Strict inclusion and exclusion criteria were applied to both groups to ensure the validity and clinical relevance of the findings.

Inclusion Criteria:

• Neonates with microbiologically confirmed Gram-negative bacterial infection occurring within the first 72 h of life.
• Positive cultures from sterile sites (e.g., blood, cerebrospinal fluid).
• Availability of complete perinatal and neonatal clinical records.
• For the control group, neonates with no clinical signs of infection and negative inflammatory markers admitted during the same period were matched for gestational age (±1 week) and birth weight (±250 g).

Exclusion Criteria:

• Neonates with mixed infections involving Gram-positive or fungal pathogens.
• Major congenital malformations or chromosomal abnormalities that could independently influence clinical evolution.
• Incomplete or missing key clinical data (e.g., absent microbiological results, missing laboratory values).
• Late-onset sepsis (defined as infection diagnosed after 72 h of life).

### 2.3. Ethical Considerations

The study was approved by the Research Ethics Committee of the Victor Babeș University of Medicine and Pharmacy, Timișoara, Romania (Approval No. 107/06.07.2020, revised in 2025) and by the Research Ethics Committee of “Louis Turcanu” Children’s Hospital, Timișoara (Approval No. 133/2025). Data were analyzed using a combination of a traditional statistical environment (Python, version 3.12) and a generative AI assistant (ChatGPT (GPT-5.2) by OpenAI). GenAI was also used to identify patterns in missing data and to suggest optimal visualizations for describing trends in clinical evolution and outcomes. The authors reviewed the final interpretation to ensure accuracy and relevance.

### 2.4. Data Analysis and Statistical Methods

All collected data were compiled and processed using Microsoft Excel and Python (version 3.12).

Descriptive statistics were used to summarize the demographic and clinical characteristics of both cohorts. Continuous variables were assessed for normality using the Shapiro–Wilk test and were expressed as means with standard deviations or medians with interquartile ranges, depending on their distribution. Comparisons between groups were conducted using the independent-samples *t*-test for normally distributed variables or the Mann–Whitney U-test for non-normally distributed variables. Categorical variables, such as respiratory distress or the need for mechanical ventilation, were analyzed using Chi-square or Fisher’s exact tests, as appropriate.

Univariate logistic regression analyses were performed to identify potential risk factors associated with Gram-negative infection. The strength of association was expressed as an odds ratio (OR). A significance threshold of *p* < 0.05 was considered statistically meaningful for all hypothesis tests. The primary outcomes evaluated were the administration of surfactant, need for respiratory support, and early neonatal morbidity.

## 3. Results

The present study analyzed and compared the clinical and perinatal characteristics of neonates with confirmed Gram-negative bacterial infections (*n*_1_ = 44) to those of a matched control group without infection (*n*_2_ = 47). During the study period, 3173 neonates were admitted to our NICU. Among them, 44 had culture-proven Gram-negative sepsis, corresponding to an incidence of 13.86 per 1000 NICU admissions. 55 cases with non-Gram-negative infection were detected in the study period.

### 3.1. Demographic and Perinatal Characteristics

The comparison between the Gram-negative-infected group and the control group reveals statistically significant differences in sex distribution and mode of delivery (Table 1). Among the infected neonates, males were predominant (79.5%), whereas in the control group, the distribution was more balanced (57.4% male). The difference in sex distribution was statistically significant (*p* = 0.0418), indicating a possible association between male sex and susceptibility to Gram-negative perinatal infection.

Regarding delivery mode, the majority of neonates in the infected group were delivered by cesarean section (C-section) (56.8%) compared to 83.0% in the control group. Natural delivery occurred more frequently in the infected group (43.2%) than in the control group (17.0%). C-section delivery was more frequent in the infection group; however, given the small sample size and the tertiary referral nature of our NICU, this variable is reported descriptively, and no inference regarding association or causality is intended.

The comparison of gestational age between the groups showed nearly identical means (34.84 vs. 34.79 weeks), with no statistically significant difference. Similarly, birth weight was higher in the infected group than in the control group, but the difference did not reach statistical significance. Most infants in both groups were moderate-to-late preterm, consistent with the case-mix of our tertiary referral NICU.

Apgar scores at both 1 and 5 minutes were lower in the infected group. At 1 minute, the infected group had a mean score of 6.52 compared to 7.00 in the control group. At 5 minutes, the scores were 7.45 and 8.02 in the infected and control groups. Neither comparison showed statistically significant differences (Table 2).

**Table 2:** Comparison of perinatal clinical parameters between Gram-negative-infected and control neonates.

Variable	Gram-Negative-Infected Group		Control Group	
	Mean	SD	Mean	SD
Gestational age (weeks)	34.84	3.77	34.79	3.04
*p*-value *	0.9409			
Birth weight (grams)	2528.41	958.70	2238.30	645.13
*p*-value *	0.0967			
Apgar score at 1 minute	6.52	2.38	7.00	1.72
*p*-value *	0.2786			
Apgar score at 5 minutes	7.45	1.97	8.02	1.26
*p*-value *	0.1090			

* Student’s *t*-test.

### 3.2. Risk Factors and Maternal Background

Analysis of perinatal risk factors between neonates infected with Gram-negative bacteria and the control group reveals several differences (Table 3). PROM was observed in 22.7% of the infected group and 10.6% of the controls; however, this difference was not statistically significant. Maternal fever occurred in two cases among infected neonates, whereas no such cases were reported in the control group; however, the association was not statistically significant.

A significant difference was observed in the presence of maternal infection, as indicated by positive cervical cultures. This risk factor was observed in three infected neonates and none in the control group, resulting in a highly significant *p*-value of 0.0001. Maternal hypertensive disorders were present in four cases among infected neonates and absent in the controls, suggesting a trend but lacking statistical significance. Diabetes Mellitus showed no meaningful association between groups, as it was present in only 1 case in the control group and there were no cases in the infected group.

### 3.3. Clinical Presentation and Laboratory Findings at Onset

Respiratory and gastrointestinal signs dominated the clinical presentation of neonates with Gram-negative bacterial infections. Specifically, respiratory distress was observed in 32 out of 44 cases (72.7%), indicating that it is a prominent early manifestation of systemic disease. Similarly, digestive symptoms such as feeding intolerance, abdominal distension, or vomiting were reported in 33 neonates (75.0%), reflecting frequent gastrointestinal involvement.

Seizures were less common but still present in a notable proportion, affecting 17 neonates (38.6%), suggesting central nervous system involvement in more severe or advanced cases. Other nonspecific symptoms, including lethargy, temperature instability, or poor perfusion, were reported in 24 of 43 infants (55.8%), indicating a diverse range of clinical signs beyond the classical presentations. The predominance of moderate and late preterm infants reflects the referral pattern of our NICU, and the added gestational-age stratification helps contextualize the intrinsic clinical vulnerability of the study population.

**Figure 1:**
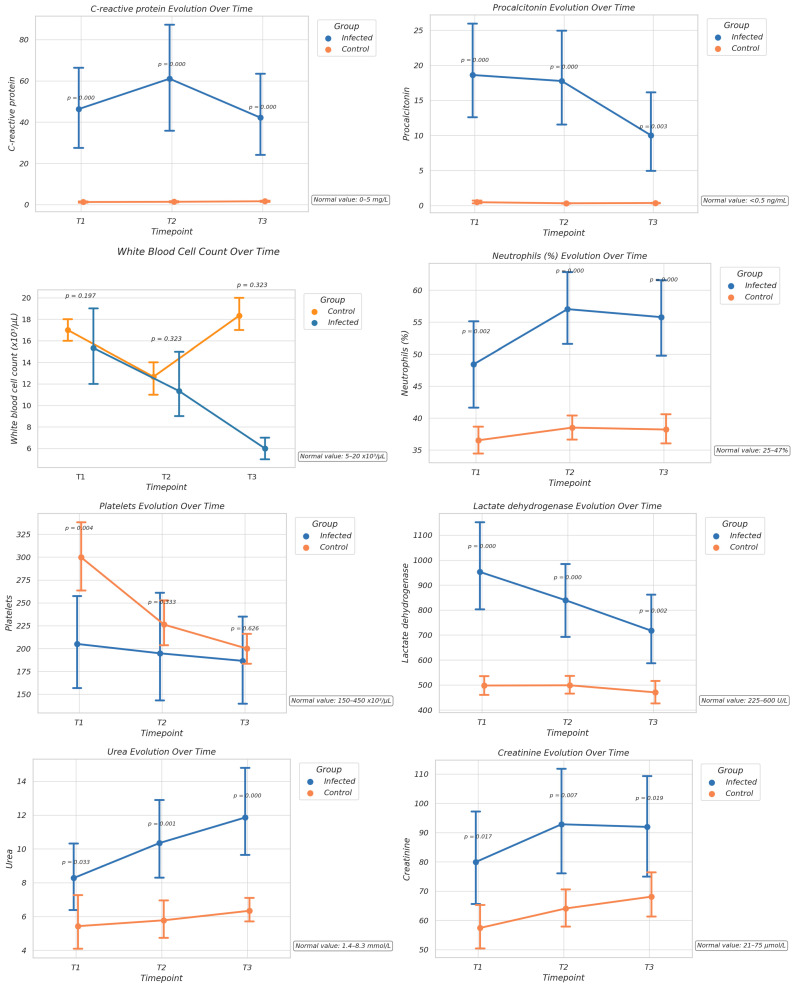

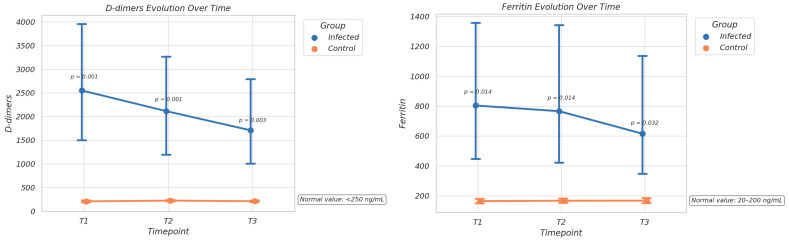
Comparative laboratory markers in infected and control groups.

To assess the dynamic inflammatory and metabolic responses associated with neonatal Gram-negative bacterial infections, a panel of laboratory biomarkers was measured at three distinct time points—T1 (initial presentation), T2 (intermediate follow-up at 48 h), and T3 (late phase at 72 h). These serial measurements allowed the evaluation of temporal trends and intergroup differences between infected neonates and uninfected controls (Figure 1).

C-reactive protein (CRP) levels were markedly elevated in the infected group compared to the controls at all time points. The highest values were observed at T1, with a gradual decline by T3, although still significantly higher than in the control group. This pattern indicates an acute-phase inflammatory response that persists over time. Procalcitonin (PCT) values mirrored the CRP trend, being significantly increased in infected neonates, particularly at T1 and T2. Although levels decreased slightly by T3, they remained above control values, supporting its role as a sensitive marker for bacterial infection and early systemic inflammation.

The white blood cell count (WBC) showed moderate elevation and notable variability over time in the infected group. Although no statistically significant differences were observed, the greatest divergence occurred at T1, suggesting a possible early leukocytic response to infection. The trend over time indicates a gradual decline in WBC among infected neonates, in contrast to relatively stable levels in the control group. However, the overlap between groups at later time points reduced its discriminatory power. Also, neutrophils (%) were significantly higher in infected neonates, especially at T1, consistent with a typical neutrophilic shift during bacterial infections. T3 observed a normalization trend in some cases.

A notable thrombocytopenia was observed in the infected group, which was most pronounced at T1 and T2, likely reflecting platelet consumption or suppression during sepsis. Platelet counts partially recovered by T3 but remained lower than in the controls.

Lactate dehydrogenase (LDH) levels were higher in the infected group at all time points, likely indicating increased tissue turnover or hypoxic damage associated with systemic infection. Urea and creatinine levels, although showing slight increases in infected neonates, particularly at T1, did not differ significantly between groups, suggesting that renal function was generally preserved during the early phases of infection in most cases. Elevated D-dimer levels in the infected group, especially at T1 and T2, reflect activation of the coagulation cascade, consistent with sepsis-associated coagulopathy. Ferritin was significantly elevated in infected neonates, particularly at T1 and T2, as it serves as both an acute-phase reactant and a potential marker of macrophage activation. Its levels showed a downward trend by T3.

### 3.4. Microbiological Profile of Gram-Negative Infections

The microbiological profile (Table 4) of the infected neonatal cohort revealed *E. coli* as the most frequently isolated pathogen, particularly in blood cultures where it accounted for 18 out of 30 positive hemocultures, including four MDR strains. This dominance underscores the central role of *E. coli* in bloodstream infections among neonates. *Klebsiella* spp. was detected less frequently, with 3 cases in blood (one MDR), and isolated once in catheter cultures and in three cases of wound culture (one MDR). *Pseudomonas* spp. was identified in five hemocultures, including two MDR strains, and in two catheter cultures. *Serratia* spp. was detected in four hemocultures, including one MDR strain.

Catheter-associated infections were polymicrobial, with seven positive cultures yielding *E. coli*, *Klebsiella* spp., and *Pseudomonas* spp.; however, none of the catheter isolates were MDR. Two urocultures were positive, both yielding *E. coli*, which were not MDR.

Overall, multidrug resistance was most commonly associated with bloodborne isolates, particularly among *E. coli*, *Pseudomonas*, and *Serratia*.

### 3.5. Interventions, Supportive Therapies, and Mortality Indicators

Among neonates infected with Gram-negative bacteria, respiratory support interventions were commonly employed (Table 5). Mechanical ventilation (VM/SIMV) was the most frequently used (33 cases) and had an average duration of 21.57 days. Free-flow oxygen was used in 21 neonates, with a mean duration of 6.67 days, while non-invasive positive pressure ventilation (nIPPV or nCPAP) was applied in 11 cases, with a mean duration of 6.36 days. High-flow nasal cannula (Optiflow) was the least frequently used, applied in 7 neonates for an average of 3.86 days. Surfactant administration was recorded in 22 neonates.

To investigate factors associated with neonatal mortality, we employed logistic regression, which allows for the estimation of odds ratios (ORs) for each predictor, providing an interpretable measure of how specific clinical or demographic factors influence mortality risk in both infected and uninfected neonates (Table 6).

In our analysis, we modeled mortality separately within the infected cohort and a matched control group. Predictors included multidrug-resistant (MDR) infection status, gestational age, birth weight, and Apgar scores at 1 and 5 min. These variables were selected based on their established relevance to neonatal outcomes and their availability in both datasets.

The results highlighted findings across both groups: gestational age emerged as a strong protective factor against mortality, with each additional week of gestation significantly reducing the odds of death (OR = 0.75 in the infected group and OR = 0.73 in the controls). Similarly, Apgar scores, particularly at 1 minute, were more strongly associated with survival in the control cohort (OR = 0.56), indicating that initial adaptation at birth remains a determinant of early outcomes, even in the absence of infection.

Interestingly, MDR infection was associated with a modest increase in mortality odds in the infected group (OR = 1.29). However, this association did not reach statistical significance, likely due to the small sample size.

Overall, this analysis highlights the importance of perinatal factors, gestational age, and immediate postnatal adaptation in determining neonatal survival. The use of regression modeling provided a structured comparative framework for assessing how these factors operate in both infected and non-infected neonates, offering valuable insights for risk stratification and clinical decision-making.

### 3.6. Immediate Clinical Outcomes

Clinical outcomes were chosen based on their clinical relevance and their ability to reflect the acute severity of neonatal illness. These outcomes serve as early indicators of disease burden and the level of supportive care required shortly after birth or upon NICU admission (Table 7).

Mechanical ventilation was required in 16.2% of infected neonates compared to only 0.15% in the control group. This difference was significant (*p* < 0.0001), indicating a strong association between Gram-negative infection and the need for advanced respiratory support. Surfactant Administration occurred more frequently in the infected group (1.07% vs. 0.02%) but this difference was not statistically significant. The mean hospitalization length was significantly longer in the infected group than in the control group; however, the difference was not statistically significant. Mortality was notably higher in the infected group (25%) than in the control group (4.3%) but the difference did not reach statistical significance.

## 4. Discussion

This study evaluated the clinical characteristics and early outcomes of neonates diagnosed with perinatal Gram-negative bacterial infections in a tertiary NICU in Timisoara, Romania. One of the major findings was a significant association between male sex and susceptibility to disease. The mode of delivery also showed a strong correlation, as natural births were significantly more frequent in the infected group, suggesting that vaginal delivery may increase exposure to maternal flora harboring Gram-negative organisms. Although gestational age and birth weight did not differ significantly between groups, Apgar scores were slightly lower in the infected group, hinting at a trend toward more compromised neonatal adaptation in these cases.

From a clinical standpoint, the infected neonates exhibited a higher incidence of respiratory distress, digestive symptoms, and neurologic involvement, reflecting the systemic nature of early-onset Gram-negative sepsis. Laboratory markers such as CRP and procalcitonin were consistently elevated in infected infants across all time points, establishing their utility as reliable indicators for early detection and monitoring. Microbiological data confirmed Escherichia coli as the predominant pathogen, with a significant burden of MDR strains, particularly in bloodstream infections. These infections were associated with higher rates of intensive respiratory support, including mechanical ventilation, and a non-significantly elevated mortality rate (25% vs. 4.3%). Overall, the findings highlight the multifaceted complexity of neonatal Gram-negative infections and underscore the need for targeted risk assessment and treatment strategies.

Recent studies on early-onset Gram-negative bacterial infections in neonates highlight a troubling scenario in NICUs, particularly concerning clinical presentation, outcomes, and trends in antibiotic resistance. Gram-negative infections in neonates are particularly severe, often resulting in poor outcomes. Research has shown a mortality rate as high as 66% in neonates suffering from Gram-negative sepsis [11], which is higher than the rate in this study. This finding highlights the life-threatening nature of these infections and aligns with global data indicating that sepsis is a significant contributor to neonatal mortality, particularly in resource-poor regions [12,13,14].

Beyond innate immune differences, environmental conditions within NICUs may amplify gender-based disparities in infection rates. The NICU setting, often involving frequent and invasive medical interventions, appears to impact male neonates more severely, leading to higher occurrences of Gram-negative bacteremia, particularly among those experiencing extended hospital stays [15]. This issue is further intensified by the more frequent application of invasive procedures such as catheter placements, which have been associated with a disproportionate increase in Gram-negative sepsis among male infants [16].

The study revealed a significant association between the mode of delivery and the risk of Gram-negative neonatal infection, with natural vaginal delivery being markedly more common among infected neonates (43.2%) compared to controls (17.0%). This finding suggests that vaginal birth may facilitate vertical transmission of Gram-negative bacteria, particularly from colonized maternal genital tracts. While cesarean sections are often perceived to reduce exposure to maternal flora, thereby lowering the risk of early-onset sepsis, our results emphasize the importance of maternal screening and intrapartum infection control in vaginal deliveries [17,18,19,20,21]. The small sample size does not allow for meaningful conclusions regarding the association between mode of delivery and infection, and these findings should be interpreted descriptively.

Antimicrobial resistance in Gram-negative bacteria further complicates treatment efforts. Multidrug resistance was observed in up to 67% of Gram-negative isolates in studies [16,22]. This alarming trend calls for ongoing reassessment of empirical antibiotic regimens. Although the World Health Organization recommends ampicillin and gentamicin as first-line therapy for suspected neonatal sepsis, the increasing prevalence of MDR organisms raises serious concerns about the continued efficacy of these guidelines [16,22].

*E. coli*, for instance, as this study shows, has been frequently identified as a dominant pathogen in higher-income countries’ settings [1,14]. On the other hand, pathogens such as *Pseudomonas* spp. and *Klebsiella* spp. are more commonly associated with late-onset infections, often linked to invasive procedures and extended NICU stays [22,23].

Specific neonatal populations are more vulnerable to Gram-negative sepsis, particularly those born prematurely or with low birth weight. These infants are at increased risk due to factors such as vertical transmission during delivery and nosocomial exposure in NICUs. Additional maternal risk factors, like PROM, further increase susceptibility [24,25]. Also, cervical colonization is strongly associated with an increased risk of neonatal Gram-negative sepsis, influencing both early-onset and late-onset infection rates [26]. Infections such as Group B *Streptococcus* (GBS) and *E. coli*, which often originate from maternal bacterial colonization, are frequent causes of neonatal sepsis [27,28]. These maternal conditions facilitate the transmission of pathogens to the neonate, making them critical targets for preventive strategies. The use of invasive monitoring devices in NICUs also increases infection risk, underscoring the importance of rigorous infection prevention protocols in both perinatal and neonatal care [29,30].

Diagnosing early-onset Gram-negative infections can be challenging, as their clinical signs often resemble those of Gram-positive infections. Accurate diagnosis relies on assessing maternal health, conducting thorough neonatal examinations, and utilizing laboratory tests such as blood cultures and biochemical markers [31,32]. Advances in biomarker research have shown that procalcitonin may aid in distinguishing between Gram-negative and Gram-positive infections early, potentially improving diagnostic precision [33].

A variety of laboratory biomarkers play a pivotal role in the diagnosis, monitoring, and prognosis of neonatal sepsis, particularly those caused by Gram-negative organisms. CRP is a widely used acute-phase marker that rises in response to inflammation. Elevated and persistent CRP levels indicate ongoing infection and may signal ineffective antibiotic therapy, necessitating timely treatment adjustments [34].

The findings of this study demonstrate that neonates with Gram-negative infections required significantly more intensive respiratory support compared to their uninfected counterparts. Mechanical ventilation was employed in 75% of infected cases, with an average duration exceeding 21 days, indicating the severity of respiratory compromise in these infants. Other forms of respiratory support, including non-invasive ventilation (nIPPV/nCPAP), free-flow oxygen, and high-flow nasal cannula (Optiflow), were also used but to a lesser extent. The frequent need for surfactant administration further underscores the importance of pulmonary dysfunction, likely exacerbated by systemic inflammation, sepsis-associated lung injury, or underlying prematurity. These results highlight the clinical burden posed by Gram-negative infections, which not only increase the risk of respiratory failure but also demand prolonged and complex supportive care in the NICU. The high reliance on invasive ventilation emphasizes the need for early detection and targeted treatment strategies to mitigate respiratory deterioration and improve outcomes.

The literature indicates that oxygen therapy is a fundamental intervention, delivered via methods such as nasal cannula, high-flow oxygen, or continuous positive airway pressure (CPAP). CPAP is particularly effective in moderate to severe distress by preventing alveolar collapse and improving gas exchange [35]. In more critical cases, mechanical ventilation may be required to manage respiratory failure, although it increases the risk of complications, such as ventilator-associated pneumonia caused by Gram-negative organisms [35]. Concurrent with respiratory support, prompt antimicrobial therapy is essential. Initiating broad-spectrum antibiotics, followed by pathogen-directed treatment based on sensitivity profiles, is critical for controlling the infection and improving respiratory outcomes [36].

Recent literature highlights a notable gap in European and Balkan data regarding the early clinical evolution and epidemiology of Gram-negative neonatal infections in NICU settings. Our search did not identify comparable studies from this region published in the last decade, with the available evidence originating predominantly from countries such as Iran, India, or Ethiopia [37,38,39]. While informative, these studies reflect distinct healthcare infrastructures, regulatory frameworks, and socioeconomic contexts, limiting their direct comparability to our setting. This scarcity of region-specific data underscores the relevance of our findings and supports the need for locally grounded research on Gram-negative neonatal infections in Europe.

Despite the valuable insights this study offers, several limitations should be acknowledged. First, the retrospective, single-center design inherently limits the generalizability of the findings, particularly in healthcare settings with differing epidemiological patterns or resource availability. This design is not intended to demonstrate whether sepsis is harmful, but rather to estimate the magnitude of the clinical impact of Gram-negative sepsis within a local NICU context. The relatively small sample size may have limited the statistical power, particularly in subgroup analyses such as mortality predictors and outcomes related to specific pathogens. Because the negative impact of Gram-negative sepsis on neonatal mortality is well established, there is no true clinical equipoise for a powered comparison of mortality between infected and non-infected infants. Accordingly, mortality differences in our study should be interpreted descriptively and not as inferential comparisons. Additionally, the inability to perform advanced pathogen typing or resistance gene profiling restricted a more nuanced understanding of the underlying microbial dynamics and mechanisms of resistance in the cohort.

Future research should aim to validate these findings through multicenter, prospective studies that include larger and more diverse neonatal populations. There is a pressing need for integrated molecular and genomic surveillance of Gram-negative pathogens to better characterize resistance patterns and inform the development of empirically tailored antibiotic guidelines for local contexts. Investigations into host immune responses, especially sex-based immunological differences and biomarker trajectories over time, could further refine risk-stratification tools. Moreover, studies evaluating the impact of standardized maternal screening protocols and targeted intrapartum prophylaxis on early-onset Gram-negative infection rates would offer valuable directions for preventive strategies. Exploring the efficacy and safety of novel therapeutic approaches, such as combination antibiotic therapies or adjunctive agents, remains an area for improving clinical outcomes in this vulnerable population.

## 5. Conclusions

This study provides a comprehensive evaluation of the clinical features, risk factors, and early outcomes associated with perinatal Gram-negative bacterial infections in neonates admitted to a tertiary care center. The findings underscore the heightened vulnerability of male neonates and those born via vaginal delivery to early-onset Gram-negative infections. *E. coli* emerged as the leading pathogen, often associated with multidrug resistance, particularly in bloodstream infections. The frequent need for intensive respiratory support, including mechanical ventilation and surfactant therapy, further evidenced the clinical burden. Mortality was higher among infected neonates, highlighting the severity of these infections. The study emphasizes the multifactorial nature of neonatal Gram-negative sepsis and the importance of timely recognition, appropriate antimicrobial therapy, and supportive care in managing affected infants.

## Figures and Tables

**Table 1:** Baseline maternal, perinatal, and neonatal characteristics in neonates with Gram-negative sepsis and matched NICU controls.

Variable		Gram-Negative-Infected Group	Control Group
Sex	Female	9	20
	Male	35	27
	*p*-value *	0.0418	
Delivery type	C-section	25	39
	Natural	19	8
	*p*-value *	0.00001	

* Chi-square test.

**Table 3:** Perinatal risk factors in the Gram-negative-infected and control neonate groups.

Variable	Gram-Negative-Infected Group		Control Group		*p*-Value *
	Present	Absent	Present	Absent	
PROM	10	34	5	42	0.1864
Maternal fever	2	42	0	47	0.4357
Maternal infection (cervical culture)	3	41	0	47	0.0001
Maternal hypertensive disorders	4	40	0	47	0.1091
Diabetes Mellitus	0	44	1	47	1.0000

* Chi-square test.

**Table 4:** Microbiological distribution and multidrug resistance of Gram-negative pathogens found in different culture sites.

Pathogen	Hemoculture (30 Positive Cases)		Uroculture (2 Positive Cases)		Wound Culture (5 Positive Cases)		Catheter Culture (7 Positive Cases)	
	Total	MDR	Total	MDR	Total	MDR	Total	MDR
*E. coli*	18	4	2	0	2	0	4	0
*Klebsiella* spp.	3	1	0	0	3	1	1	0
*Pseudomonas* spp.	5	2	0	0	0	0	2	0
*Serratia* spp.	4	1	0	0	0	0	0	0

**Table 5:** Intervention usage in the Gram-negative-infected group.

Intervention	Used	Mean Day Used	Not Used
Free-flow oxygen	21	6.67	23
High-flow nasal cannula (Optiflow)	7	3.86	37
nIPPV or nCPAP	11	6.36	33
Mechanical ventilation (VM/SIMV)	33	21.57	11
Surfactant administration	22	-	22

**Table 6:** Mortality predictors between the control and infected groups.

Predictor	Gram-Negative-Infected Group		Control Group	
	Coefficient	OR	Coefficient	OR
MDR infection	0.2547	1.2901	0.0	1.0
Gestational age	−0.2853	0.7517	−0.3143	0.7303
Birth weight	0.0805	1.0838	−0.209	0.8114
Apgar 1 min	0.1227	1.1305	−0.572	0.5644
Apgar 5 min	−0.0392	0.9615	−0.3604	0.6974

**Table 7:** Comparison of Immediate Clinical Outcomes.

Outcome	The Gram-Negative Infected Group	Control Group	*p*-Value
Use of mechanical ventilation	16.2%	0.15%	<0.0001
Surfactant administration	1.07%	0.02%	0.2645
Mean length of hospitalization in NICU (days)	32.34	26.02	0.1112
Mortality	25%	4.3%	1.0000

## Data Availability

The data supporting this study are available from the corresponding author upon request. However, due to ethical considerations, they are not publicly accessible.

## Abbreviations

The following abbreviations are used in this manuscript:

NICU	Neonatal Intensive Care Unit
PROM	Premature rupture of membranes
CRP	C-Reactive Protein
PCT	Procalcitonin
WBC	White blood cell
LDH	Lactate dehydrogenase
MDR	Multidrug resistant
nIPPV	Non-invasive positive pressure ventilation
nCPAP	Nasal continuous positive airway pressure
VM/SIMV	Volume-mode/Synchronized intermittent mandatory ventilation
GBS	Group B *Streptococcus*
TNF-α	Tumor necrosis factor-alpha
IL-6	Interleukin-6
EPI	Efflux pump inhibitor

## References

[1] Hallmaier-Wacker L.K., Andrews A., Nsonwu O., Demirjian A., Hope R.J., Lamagni T., Collin S.M. (2022). Incidence and Aetiology of Infant Gram-Negative Bacteraemia and Meningitis: Systematic Review and Meta-Analysis. Arch. Dis. Child..

[2] Chan G.J., Lee A.C., Baqui A.H., Tan J., Black R.E. (2015). Prevalence of Early-Onset Neonatal Infection among Newborns of Mothers with Bacterial Infection or Colonization: A Systematic Review and Meta-Analysis. BMC Infect. Dis..

[3] Yu X., Chen H., Chen G. (2024). Analysis of Risk Factors and Complications of Neonatal Infection in 500 Pregnant Women with Premature Rupture of Membranes. Research Square.

[4] Siefker D.T., Adkins B. (2017). Rapid CD8+ Function Is Critical for Protection of Neonatal Mice from an Extracellular Bacterial Enteropathogen. Front. Pediatr..

[5] Celik I.H., Hanna M., Canpolat F.E. (2022). Mohan Pammi Diagnosis of Neonatal Sepsis: The Past, Present and Future. Pediatr. Res..

[6] Morris S., Cerceo E. (2020). Trends, Epidemiology, and Management of Multi-Drug Resistant Gram-Negative Bacterial Infections in the Hospitalized Setting. Antibiotics.

[7] Domalaon R., Idowu T., Zhanel G.G., Schweizer F. (2018). Antibiotic Hybrids: The Next Generation of Agents and Adjuvants against Gram-Negative Pathogens?. Clin. Microbiol. Rev..

[8] Kobayashi S.D., DeLeo F.R. (2018). Re-Evaluating the Potential of Immunoprophylaxis and/or Immunotherapy for Infections Caused by Multidrug Resistant *Klebsiella pneumoniae*. Future Microbiol..

[9] Daniel C.P., Sittig K.M., Wagner M.J., Cade C., Chriss W. (2024). Antibiotic Treatment Practices and Microbial Profile in Diabetic Foot Ulcers: A Retrospective Cohort Study. Cureus.

[10] Daoud Z., Dropa M. (2023). Editorial: The Global Threat of Carbapenem-Resistant Gram-Negative Bacteria, Volume II. Front. Cell. Infect. Microbiol..

[11] Nafea S., Gaafar M., Fakhr A., Mokhtar W. (2019). Toll Like Receptor Type Four Gene Polymorphism in Neonatal Sepsis in Zagazig University Children’s Hospital. Zagazig Univ. Med. J..

[12] Wen S.C.H., Ezure Y., Rolley L., Spurling G., Lau C.L., Riaz S., Paterson D.L., Irwin A.D. (2021). Gram-Negative Neonatal Sepsis in Low- and Lower-Middle-Income Countries and WHO Empirical Antibiotic Recommendations: A Systematic Review and Meta-Analysis. PLoS Med..

[13] Al Abdullatif M. (2019). Microbiology of Neonatal Gram-Negative Sepsis in A Level III Neonatal Intensive Care Unit (NICU). A Single Center Experience. GJPNC.

[14] Hallmaier-Wacker L.K., Andrews A., Hope R., Demirjian A., Lamagni T.L., Collin S.M. (2023). Incidence of Infant Gram-Negative Invasive Bacterial Infections in England, 2011–2019: An Observational Study Using Population-Wide Surveillance Data. Arch. Dis. Child..

[15] Lee P., Sin E., Yip K.-T., Ng K. (2025). A 10-Year Study of Neonatal Sepsis from Tuen Mun Hospital, Hong Kong. Pathogens.

[16] Sands K., Carvalho M.J., Portal E., Thomson K., Dyer C., Akpulu C., Andrews R., Ferreira A., Gillespie D., Hender T. (2021). Characterization of Antimicrobial-Resistant Gram-Negative Bacteria That Cause Neonatal Sepsis in Seven Low- and Middle-Income Countries. Nat. Microbiol..

[17] Negrini R., Da Silva Ferreira R.D., Guimarães D.Z. (2021). Value-Based Care in Obstetrics: Comparison between Vaginal Birth and Caesarean Section. BMC Pregnancy Childbirth.

[18] Chu D.M., Ma J., Prince A.L., Antony K.M., Seferovic M.D., Aagaard K.M. (2017). Maturation of the Infant Microbiome Community Structure and Function across Multiple Body Sites and in Relation to Mode of Delivery. Nat. Med..

[19] Kuang Y.-S., Li S.-H., Guo Y., Lu J.-H., He J.-R., Luo B.-J., Jiang F.-J., Shen H., Papasian C.J., Pang H. (2016). Composition of Gut Microbiota in Infants in China and Global Comparison. Sci. Rep..

[20] Darvish S., Etemad K., Mosaheb A., Yazdanpanah G. (2017). Comparing Maternal and Neonatal Side Effects of Natural Vaginal Delivery under Neuro-Axial Analgesia with Usual Vaginal Delivery and Cesarean Section: A Primary Single Center Study. J. Obstet. Gynecol. Cancer Res..

[21] Wu S.-W., Zhang W.-Y. (2021). Effects of Modes and Timings of Delivery on Feto-Maternal Outcomes in Women with Severe Preeclampsia: A Multi-Center Survey in Mainland China. Int. J. Gen. Med..

[22] Chiusaroli L., Liberati C., Caseti M., Rulli L., Barbieri E., Giaquinto C., Donà D. (2022). Therapeutic Options and Outcomes for the Treatment of Neonates and Preterms with Gram-Negative Multidrug-Resistant Bacteria: A Systematic Review. Antibiotics.

[23] Flannery D.D., Chiotos K., Gerber J.S., Puopolo K.M. (2022). Neonatal Multidrug-Resistant Gram-Negative Infection: Epidemiology, Mechanisms of Resistance, and Management. Pediatr. Res..

[24] Kurma V.R., Raju M., Manchu T., Manchu K. (2019). Neonatal Sepsis: Clinical Spectrum, Bacteriological Profile and Antibiotic Sensitivity Patterns in Neonatal Intensive Care Unit in a Tertiary Care Hospital. Int. J. Contemp. Med. Res..

[25] Masanja P.P., Kibusi S.M., Mkhoi M.L. (2020). Predictors of Early Onset Neonatal Sepsis among Neonates in Dodoma, Tanzania: A Case Control Study. J. Trop. Pediatr..

[26] Abd Alazem E., Abdel Ghany E., Zaky S., Abd Elhady M. (2022). Thrombocytopenia Is More Frequent in Gram Negative Neonatal Septicemia. Pediatr. Sci. J..

[27] Shobowale E., Solarin A., Elikwu C., Onyedibe K., Akinola I., Faniran A. (2017). Neonatal Sepsis in a Nigerian Private Tertiary Hospital: Bacterial Isolates, Risk Factors, and Antibiotic Susceptibility Patterns. Ann. Afr. Med..

[28] Salahi M., Moghadam A.G., Mousavizadeh A., Marashifard M., Taghavi S.J., Rezanejad M., Moradi S., Khoramrooz S.S. (2020). Neonatal Sepsis in Southwest of Iran; Prevalence of Microbial Pathogens, Risk Factors, Clinical Manifestations and Laboratory Findings. Research Square.

[29] Tang S., Li M., Chen H., Ping G., Zhang C., Wang S. (2017). A Chronological Study of the Bacterial Pathogen Changes in Acute Neonatal Bacterial Conjunctivitis in Southern China. BMC Ophthalmol..

[30] Singh A., Garg A.K. (2019). Neonatal Sepsis and Identification of Risk Factors: Study in Kmmc and Hospital Mathura. Int. J. Med. Biomed. Stud..

[31] Kent A., Kortsalioudaki C., Monahan I.M., Bielicki J., Planche T.D., Heath P.T., Sharland M. (2016). Neonatal Gram-Negative Infections, Antibiotic Susceptibility and Clinical Outcome: An Observational Study. Arch. Dis. Child. Fetal Neonatal Ed..

[32] Akya A., Rostamian M., Rezaeian S., Ahmadi M., Janatolmakan M., Sharif S.A., Ahmadi A., Weisi S., Chegene Lorestani R. (2020). Bacterial Causative Agents of Neonatal Sepsis and Their Antibiotic Susceptibility in Neonatal Intensive Care Units (NICUs) and Neonatal Wards in Iran: A Systematic Review. Arch. Pediatr. Infect. Dis..

[33] Thomas R., Ondongo-Ezhet C., Motsoaledi N., Sharland M., Clements M., Velaphi S. (2022). Incidence and All-Cause Mortality Rates in Neonates Infected With Carbapenem Resistant Organisms. Front. Trop. Dis..

[34] El-Nahhal Y.Z., Al_Shareef A.T., Alagha M.R. (2019). Measurements of C-Reactive Protein for Successful Management and Follow-Up Treatment of Neonatal Sepsis and Nosocomial Infection. Health.

[35] Karacanoglu D., Tanyeri Bayraktar B., Bayraktar S., Meric Z., Hepokur M. (2018). Retrospective Evaluation of Culture Proven Neonatal Sepsis Cases in Neonatal Intensive Care Unit. Bezmialem Sci..

[36] Hussain M.A., Akram S., Khan M.A., Nawaz S., Ali S., Amir M. (2023). Neonatal Sepsis; Incidence and Microbiological Profile along with Antibiotic Sensitivity of Causative Microorganisms. Life Sci..

[37] Solomon S., Akeju O., Odumade O.A., Ambachew R., Gebreyohannes Z., Van Wickle K., Abayneh M., Metaferia G., Carvalho M.J., Thomson K. (2021). Prevalence and Risk Factors for Antimicrobial Resistance among Newborns with Gram-Negative Sepsis. PLoS ONE.

[38] Aletayeb S.M.H., Dehdashtian M., Malakian A., Aramesh M.R., Kouti L., Aletayeb F. (2023). Frequency, Bacteriological Profile, and Outcome of Neonatal Sepsis with Carbapenem-Resistant Gram-Negative Bacteria at the Tertiary Neonatal Intensive Care Unit, Ahvaz, Iran. Jundishapur J. Microbiol..

[39] Pataskar A., Chandel A., Chauhan V., Jain M. (2023). Gram-Negative Late Onset Neonatal Sepsis in a Tertiary Care Center From Central India: A Retrospective Analysis. Clin. Med. Insights Pediatr..

